# Separation Performance of Capillary Gas Chromatography Based on Monohydroxycucurbit[7]Uril Incorporated Into Sol–Gels as the Stationary Phase

**DOI:** 10.3389/fchem.2020.00031

**Published:** 2020-02-05

**Authors:** Jing He, Jingfeng Ran, Jianmei Yao, Lingxue Zhang, Shasha Wang, Yuan Wang, Nan Dong

**Affiliations:** ^1^School of Chemistry and Chemical Engineering, Guizhou University, Guiyang, China; ^2^Resource and Environmental Engineering College, Guizhou University, Guiyang, China; ^3^Key Laboratory of Macrocyclic and Supramolecular Chemistry of Guizhou Province, Guiyang, China

**Keywords:** monohydroxycucurbit[7]uril, stationary phase, sol–gel coating, capillary gas chromatography, separation performance

## Abstract

A novel monohydroxycucurbit[7]uril-based stationary phase for capillary gas chromatography (GC) has been produced. For this, a capillary column coating was made using a sol–gel technique, incorporating synthesized monohydroxycucurbit[7]uril [(OH)Q[7]] and hydroxy-terminated poly(dimethylsiloxane) (OH-PDMS) into the sol–gel network through hydrolysis and polycondensation and chemical sol–gel grafting to the inner wall of a fused-silica tube. The preparation method may produce a coating with greater integrity, which gives the prepared column a higher separation efficiency and better selectivity toward analytes than a reported stationary phase based on neat cucurbit[n]urils (Q[n]s). The prepared (OH)Q[7]/PDMS column had 3,225 theoretical plates per meter determined using naphthalene at 120°C and exhibited a weakly polar nature. The (OH)Q[7]/PDMS column has high resolution over a broad spectrum of analytes with symmetrical peak shapes and exhibited better separation performance than commercial capillary columns and reported columns based on neat Q[n]s that failed to resolve some critical analytes. Moreover, the column also showed good thermal stability up to 300°C and separation repeatability with relative standard deviation values in the range of 0.01–0.11% for intraday, 0.11–0.32% for interday and 0.29–0.58% for column-to-column. In addition, the energy effect on the retention of analytes on the (OH)Q[7]/PDMS stationary phase was investigated. The results indicated that retention on the column was determined mainly by the enthalpy change. As demonstrated, the proposed coating method can address some disadvantages that exist with the reported Q[n]s columns and combine the full advantages of (OH)Q[7] with the sol–gel coating method while achieving outstanding GC separation performance.

## Introduction

Capillary gas chromatography (GC) is a high-resolution analytical method. A selective stationary phase and an efficient column preparation method are the two main factors for GC column separation with high resolution (Luong, 2002). Cucurbit[n]urils (Q[n], *n* = 5–8, 10) possess excellent molecular-recognition properties toward analytes of different kinds, which is attributed to their hydrophobic cavity and two identical carbonyl-laced portals (Freeman et al., 1981; Lee et al., 2003). At the same time, the structure of Q[n]s is chemically inert and shows excellent chemical and thermal stability (Liu et al., 2005). The unique structures and molecular-recognition properties of Q[n]s have attracted attention for use as a chromatography stationary phase. Hydrophilic-interaction chromatography (Liu et al., 2004), capillary electrophoresis (Xu et al., 2004; Wei and Feng, 2008), liquid chromatography (Cheong et al., 2008) and packed GC (Li et al., 2007, 2012) were reported to use parent Q[n]s or their derivative forms as the stationary phase. Qi and co-workers have undertaken relatively comprehensive research on Q[n]s as stationary phases for capillary GC. They prepared capillary columns based on Q[7], Q[8], binary Q[7]–Q[8] and a Q[8]–Cd coordination complex using a statically coated method using suspensions in dichloromethane (Sun et al., 2014; Zhang et al., 2014). To improve the solubility of Q[n]s, guanidinium-based ionic liquids were introduced to prepare Q[6]-GBIL and Q[7]-SG capillary GC columns using the sol–gel method (Wang et al., 2014a,b) (GBIL and SG are the same ionic liquid, namely *N*′′,*N*′′-(6′,6′-dihydrox)dihexyl-*N*,*N*,*N*′,*N*′-tetramethylguanidiniumbis(trifluoromethylsulfonyl)imide). Briefly, these studies demonstrate the potential for using Q[n]s in chromatographic separation, while a capillary GC column based on Q[n]s exhibited higher separation resolution among the abovementioned different chromatographic methods. However, there are some disadvantages in the preparation method for capillary GC column only using parent Q[n]s. Owing to the low solubility of Q[n]s in organic solvents, only a small amount of Q[n] adhered on the inner wall of the capillary tube, which leads to instability of the stationary phase, easy loss, and poor repeatability. Even though ionic liquids and the sol–gel coating method were simultaneously employed to prepare capillary GC columns, parent Q[n]s are encapsulated rather than incorporated into the sol–gel network structure (Liang et al., 2004), which may lead to a decrease in the selectivity of Q[n]s toward analytes, thereby influencing GC separation performance. Therefore, Q[n]s derivatization may be an effective way to overcome the problems associated with the preparation of capillary GC column-based parent Q[n]s. Qi et al. reported perhydroxycucurbit[6]uril ([OH]_12_Q[6]) as a stationary phase for capillary GC (Sun et al., 2015). Though the column efficiency based on [OH]_12_Q[6] increased up to 2380 theoretical plates per meter compared with other capillary columns based on parent Q[n]s, only a small amount of [OH]_12_Q[6] stuck to the inner wall of the capillary tube due to the low solubility of [OH]_12_Q[6] and the statically coated method, which also showed the aforementioned shortcomings. Furthermore, it is quite challenging to modify Q[n]s, especially the active group bond on the structure of Q[n]s due to their chemical inertness.

Among the family members, Q[7] has a larger dimension in terms of diameter and cavity, and easily interacts with a wide range of analytes. To date, there are only a few literature reports on the derivatization of Q[7] (Jon et al., 2003; Kim et al., 2007; Lim et al., 2008; Vinciguerra et al., 2012; Ahn et al., 2013; Lau et al., 2014; Ayhan et al., 2015). Our research group successfully synthesized monohydroxycucurbit[7]uril [(OH)Q[7]] in 2018 and found that Q[7] and (OH)Q[7] have very similar structures and properties (Dong et al., 2008). Here, we report the first example of using (OH)Q[7] as a stationary phase for capillary GC separations. Meanwhile, the sol–gel coating method was adopted for the capillary column fabrication, in which (OH)Q[7] was incorporated into the sol–gel network together with poly(dimethylsiloxane) (OH-PDMS) and became a part of the coating. In this work, chromatographic parameters including column efficiency and McReynolds' constants, separation performance of compounds with different polarity and structure, and solvent and thermal stability for the prepared (OH)Q[7]/PDMS capillary column were investigated separately. Notably, to highlight the advantages of the prepared (OH)Q[7]/PDMS column, the separation performance of the (OH)Q[7]/PDMS capillary column was compared with that of the reported columns based on Q[n]s. Finally, energy effects on the retention of analytes were also investigated to obtain a better understanding of their retention behaviors and separation mechanism.

## Experimental

### Chemicals and Equipment

Methyltrimethoxysilane (MTMOS, 98%), poly (methylhydrosiloxane) (PMHS, 99%), trifluoroacetic acid (TFA, 95%), hydroxy-terminated poly(dimethylsiloxane) (OH-PDMS, 99%) and 3-(2-cyclooxypropoxyl)propyltrimethoxysilane (KH-560, 97%) were purchased from Alatin Reagents Co. (Shanghai, China). (OH)Q[7] was synthesized and purified according to our previously published method (Dong et al., 2008). Dichloromethane and the rest of the chemicals used in this work were purchased from Alatin Reagents Co. (Shanghai, China). All the chemicals were at least of analytical grade and dissolved in dichloromethane. Untreated fused-silica capillary tubing (0.25 mm, i.d.) was purchased from Yongnian Ruifeng Chromatogram Apparatus Co., Ltd. (Hebei, China). A commercial SE-54 capillary column (30 m × 0.25 mm, i.d., 0.25 μm film thickness), OV-1701 capillary column (30 m × 0.25 mm, i.d., 0.25 μm film thickness) and FFAP capillary column (30 m × 0.25 mm, i.d., 0.25 μm film thickness) were from Dalian Huawushuo Institute (Dalian, China) for comparison.

An Agilent 6890 gas chromatograph equipped with split/splitless injectors and flame ionization detectors and operated at 300°C was used. Nitrogen of high purity (99.999%) was used as the carrier gas, and 1 mL/min velocity was used. The split flow was adjusted to 30 mL/min. A Hitachi X-650 (Tokyo, Japan) scanning electron microscope (SEM) was used for the morphological observation of the coated capillary columns. Infrared (IR) spectra were recorded on a VERTEX70 Fourier-transform IR spectrometer (Bruker Optics, Karlsruhe, Germany) in the 400–4,000 cm^−1^ region.

### Capillary Column Preparation

The capillary column based on (OH)Q[7] was prepared using the sol–gel coating method. Prior to coating, a capillary column (10 m × 0.25 mm, i.d.) was successively pretreated with 1 mol/L NaOH and 0.1 mol/L HCl, rinsed with water and dried overnight at 120°C under nitrogen. After this, 30 μL MTMOS, 50 μL PMHS, 300 μL OH-PDMS and 400 μL CH_2_Cl_2_ were added to a vial and mixed well. Then, 20 mg (OH)Q[7], 300 μL TFA, 100 μL KH-560, and 300 μL CH_2_Cl_2_ were added and sonicated for 5 min and centrifuged for 9 min (12,000 r/min). The clear top portion of the resulting sol solution was pumped into the pretreated capillary column and stayed for 30 min at room temperature. After the excess sol solution was expelled from the column, the column was stood for 1 h under an atmosphere of nitrogen. The column was conditioned from 40 to 150°C at a rate of 1°C/min and held at 150°C for 6 h, then the temperature was increased to 250°C at a rate of 1°C/min and held at the final temperature for 4 h. Using the same coating method, the blank sol–gel column without (OH)Q[7] was also prepared for comparison.

### McReynolds' Constants

McReynolds' constants are often used to characterize the polarity of a GC stationary phase and its molecular interaction with analytes. Benzene, *n*-butanol, 2-pentanone, nitropropane and pyridine represented as π-electron interaction (X′), H-bond donor and acceptor interaction (Y′), H-bond acceptor interaction (Z′), dipole interaction (U′), and strong H-bond acceptor interaction (S′), respectively. They were used as probe compounds to determine the differences in the retention indices on the given stationary phase and squalane. The average of the differences is used for the polarity estimation. The higher the value of the average polarity, the higher the polarity of the stationary phase. In this work, McReynolds' constants of the (OH)Q[7]/PDMS column were determined at 120°C.

## Results and Discussion

### Possible Structure of the Coating

**Figure 1:**
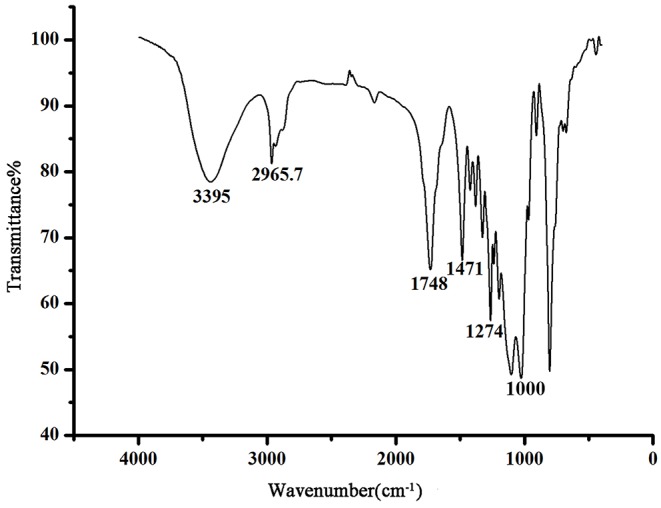
FT-IR spectrum of the (OH)Q[7]/PDMS coating.

The chemical ingredients used to create the sol–gel (OH)Q[7]/PDMS coating are presented in Table S1 in the supporting information. Being characterized by the interactions of hydroxyl groups during all steps, (OH)Q[7] and OH-PDMS are used as the components of the coating stationary phase to form chemical bonds between the coating and the capillary inner surface (Zeng et al., 2001). The sol–gel process starts with the catalytic hydrolysis of the sol–gel precursors and other sol–gel active ingredients in the coating solution, followed by polycondensation of the hydrolyzed products into a sol–gel network. In the process of sol–gel reaction, ring-opening polymerization takes place between KH-560 and (OH)Q[7] catalyzed by TFA, which is used to further connect (OH)Q[7] with other polymers (Zeng et al., 2001). Based on this, (OH)Q[7] incorporates into the sol–gel network and becomes one part of the coating. A simplified scheme of the sol–gel (OH)Q[7]/PDMS coating on the fused-silica capillary inner wall is presented in Scheme S1. The structure of the coating was further identified using FT-IR and shown in Figure 1. An intense band observed at about 3,395 cm^−1^ can indicate the axial stretching vibrations of O–H of PDMS, and the vibration bands at 2,965, 1,748, 1,471, and 1,274 cm^−1^ are the stretching vibration of C–H, C=O, C–N, and C–O of (OH)Q[7], respectively. The stretching vibration of C–O–C appeared at 1,000 cm^−1^, suggesting the formation of a new chemical bond and showing (OH)Q[7] was anchored to the sol–gel network. All characteristic IR absorption bands in the sol–gel-derived (OH)Q[7]/PDMS coating confirmed that the bonding of (OH)Q[7] formed an organic–inorganic copolymer coating (Shu et al., 2014).

### Characterization of the Capillary Column

**Figure 2:**
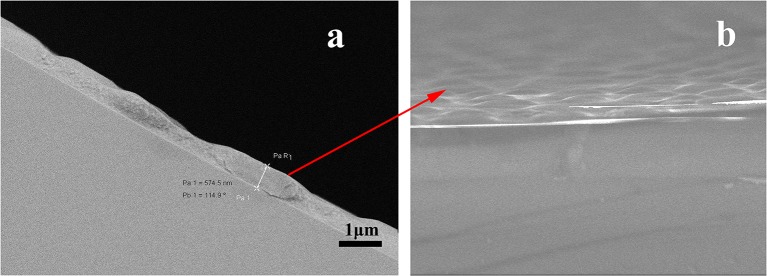
Scanning electron microscopic images of a sol-gel (OH)Q[7]/PDMS coated fused- silica capillary. **(a)** Cross-sectional view, magnification 1,200×; **(b)** surface view of the fine structures on the sol-gel (OH)Q[7]/PDMS coating, magnification, 10,000×.

Column efficiency of the as-prepared (OH)Q[7]/PDMS column was measured using *n*-dodecane and naphthalene as test solutes at 120°C, and the determined column efficiency was 2,702 and 3,225 theoretical plates/m for *n*-dodecane and naphthalene, respectively. The prepared column has higher column efficiency than those of the published columns based on Q[n]s (*n* = 6–8; Sun et al., 2014, 2015; Wang et al., 2014a,b; Zhang et al., 2014), whose column efficiency ranged from 1,060 plates/m to 2,380 plates/m, in which *n*-dodecane or naphthalene was used as test analyte and determined at 120°C. The higher column efficiency may be attributed to the uniformity and integrity of the coating on the inner wall of the capillary column prepared using the sol–gel method (Shende et al., 2003). The morphology of the (OH)Q[7]/PDMS coating on the capillary inner wall has been characterized using a scanning electron microscope (SEM) (Figure 2). As can be seen from Figure 2a, the coating is uneven, having a rough thickness of ≈0.8 μm (magnification of 1,200). The SEM image in Figure 2b shows that the coating surface is rough and wrinkled, which can increase the surface area of the coating and enhance the solute/stationary phase interactions during GC separations. A thicker layer and a roughened and wrinkled surface make the prepared (OH)Q[7]/PDMS column possess enhanced sample load and better retention toward analytes (Shende et al., 2003).

Table S2 lists the McReynolds constants of the (OH)Q[7]/PDMS column and commercial columns for comparison. In general, the stationary phase in gas chromatography can be divided into non-polar, weakly-to-moderately polar or highly polar when average polarity is 1–100, 100–400, or over 400, respectively (Sun et al., 2014). The (OH)Q[7]/PDMS column had an average polarity of 143 and can be classified as weakly-to-moderately polar nature in GC separation. As shown in Table S2, the (OH)Q[7]/PDMS stationary phase exhibits a higher value of Y′, suggesting its stronger H-bonding interaction with analytes. This can be due to the selective interactions between the carbonyl groups at portals or methine/methene protons of (OH)Q[7] and analytes via H-bonding.

### Solvent Stability and Thermal Stability of the Sol–Gel (OH)Q[7]/PDMS Column

The solvent stability was measured by the change of the solute retention times in five replicates before and after the column was rinsed with methylene chloride (Table S3). The relative standard deviation (RSD) values in retention time for 10 solutes were <0.81%, indicating that this column has excellent solvent stability. Strong immobilization and structural integrity are important properties of the prepared sol–gel (OH)Q[7]/PDMS stationary phase, which helps to maintain good reproducibility of retention time (Delahousse et al., 2013).

Strong chemical immobilization is also helpful for the prepared sol–gel (OH)Q[7]/PDMS column to enhance its thermal stability. The thermal stability of the (OH)Q[7]/PDMS column was evaluated by GC separation of mixtures containing esters, alkanes, and aldehydes after the column aged for 4 h at 210, 240, 280, and 300°C, respectively. As can be seen in Table 1, the RSD values of the analytes for the retention factor (*k*) range from 3.35 to 5.13%, indicating that the (OH)Q[7]/PDMS column has good thermal stability and separation repeatability at the specified temperature. Importantly, the separation factor (α) remained almost unchanged with the RSD <1.77%, suggesting that the column still possessed excellent separation performance for the analytes, in which no significant drift or reduction in resolution was observed in the process of separation. The highest operating temperature of the sol–gel (OH)Q[7]/PDMS column (300°C) is higher than that of the reported columns based on Q[n]s (Sun et al., 2014, 2015; Wang et al., 2014a,b; Zhang et al., 2014) (200–280°C) and commercial medium polar OV-1701 column that is recommended to be used below 270°C.

**Table 1:** Repeatability of retention factor and separation factor of the analytes in the mixture after the column was conditioned up to the indicated temperatures for 4 h.

**Analyte**	***k***					**α**				
	**210^**°**^C**	**240^**°**^C**	**280^**°**^C**	**300^**°**^C**	**RSD (%)**	**210^**°**^C**	**240^**°**^C**	**280^**°**^C**	**300^**°**^C**	**RSD (%)**
Decane	10.58	10.73	10.80	11.80	5.06	–	–	–	–	–
Undecane	13.88	14.09	14.23	15.52	5.13	1.31	1.31	1.32	1.32	0.44
Nonanal	15.03	15.23	15.28	16.19	3.35	1.08	1.08	1.07	1.04	1.77
Tridecane	20.14	20.48	20.71	22.53	5.10	1.34	1.34	1.35	1.39	1.76
Methyl decanoate	21.67	22.01	22.19	23.72	4.05	1.07	1.07	1.07	1.05	0.94
Tetradecane	23.02	23.41	23.70	25.74	5.06	1.06	1.06	1.06	1.08	0.94
Methyl undecanoate	24.47	24.83	25.09	26.87	4.21	1.06	1.06	1.05	1.04	0.91
Mehtyl dodecanoate	27.51	27.96	27.83	29.85	3.74	1.12	1.12	1.10	1.11	0.86

### Reproducibility Characteristics of the Sol–Gel (OH)Q[7]/PDMS Column

Reproducibility is an important feature for any newly developed technique. Separation repeatability of the (OH)Q[7]/PDMS column concerning intraday, interday, and column-to-column repeatability was examined by separations of mixtures. The RSD in retention times of the analytes in the mixtures was used for the evaluation (Table 2). RSD values of the analytes are in the range of 0.01–0.11% for intraday, 0.11–0.32% for interday, and 0.29–0.58% for column-to-column. These low RSD values show that the developed sol–gel method for the preparation of the (OH)Q[7]/PDMS column is indeed reliable and highly reproducible.

**Table 2:** Separation repeatability of (OH)Q[7]/PDMS capillary column in retention times (*t*_R_, min) for separation of the indicated analytes.

**Analyte**	**Intra-day (*****n*** **=** **6)**		**Inter-day (*****n*** **=** **6)**		**Column- to- column (*****n*** **=** **3)**	
	**Mean**	**RSD%**	**Mean**	**RSD%**	**Mean**	**RSD%**
Decane	6.60	0.11	6.42	0.24	6.71	0.48
Undecane	8.58	0.06	8.37	0.20	8.72	0.38
Nonanal	9.21	0.06	8.99	0.32	9.32	0.58
Tridecane	12.31	0.03	12.10	0.16	12.55	0.41
Methyl decanoate	13.19	0.04	12.96	0.26	13.41	0.36
Tetradecane	14.03	0.01	13.81	0.12	14.32	0.29
Methyl undecanoate	14.86	0.02	14.61	0.13	15.13	0.36
Mehtyl dodecanoate	16.44	0.02	16.18	0.11	16.76	0.46

### Separation Performance

The separation performance of the (OH)Q[7]/PDMS column was evaluated by GC separation of different analytes of great variety, including non-polar analytes, polar analytes, Grob mixture consisting of 12 test analytes, mixtures with similar boiling point and geometric isomers.

#### Separation of Non-Polar Analytes, Medium Polar Analytes, Polar Analytes, and Grob Mixture

**Figure 3:**
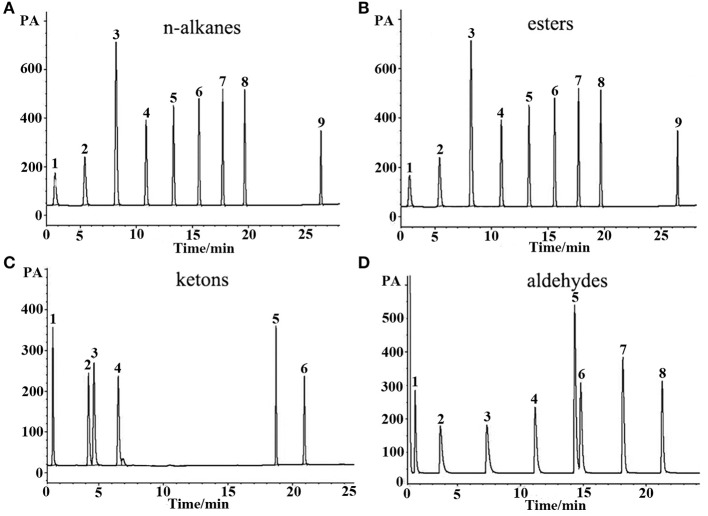
GC separations of **(A)** *n*-alkanes, **(B)** esters, **(C)** ketons, and **(D)** aldehydes on the (OH)Q[7]/PDMS column. Peaks for (a): (1) *n*-heptane, (2) *n*-octane, (3) *n*-nonane, (4) *n*-decane, (5) *n*-undecane, (6) *n*-dodecane, (7) *n*-tridecane, (8) *n*-tetradecane, (9) *n*-octodecane. Peaks for (b): (1) butyl acetate, (2) methyl heptanoate, (3) ethyl hexanoate, (4) methyl benzoate, (5) methyl caprylate, (6) methyl salicylate, (7) methyl decanoate, (8) methyl undecanoate, (9) methyl dodecanoate. Peaks for (c): (1) 2- pentanone, (2) cyclohexanone, (3) 2- heptanone, (4) 2-octanone, (5) 2-benzophenone, (6) 2- acetophenone. Peaks for (d): (1) butyraldehyde, (2) valeraldehyde, (3) hexaldehyde, (4) heptaldehyde, (5) benzaldehyde, (6) caprylaldehyde, (7) nonanal, (8) decanal. Temperature program: 40°C (1 min) to 250°C at 5°C/min.

**Figure 4:**
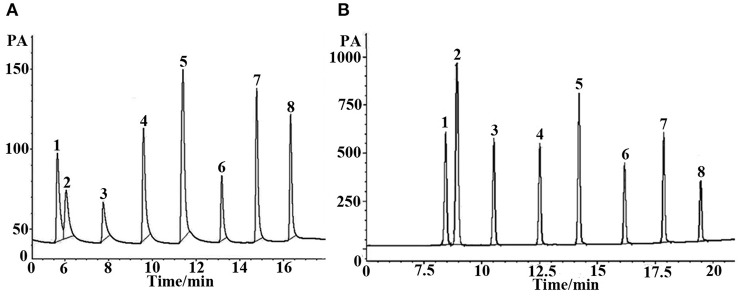
GC separations of alcohols on **(A)** the (OH)Q[7]/PDMS and **(B)** the sol-gel blank PDMS column. Peaks: (1) 1- hexanol, (2) cyclohexanol, (3) 1-heptanol, (4) 1-octanol, (5) phenethyl alcohol, (6) 1- decanol, (7) 1- undecanol, (8) 1- dodecanol. Temperature program: 40°C (1 min) to 220°C at 8°C/min.

*n*-Alkanes are typical non-polar analytes, esters, aldehydes, and ketones are low to medium polarity compounds containing oxygen atom without active hydrogen. As mentioned above, the (OH)Q[7]/PDMS stationary phase was weakly-to-moderately polar, and thus non-polar and medium polar analytes were easily separated according to “like dissolves like.” As shown in Figure 3, all the above analytes containing *n*-alkanes, esters, ketones, and aldehydes were baseline resolved and the peaks were sharp and symmetrical. For typical polar alcohol compounds, the GC chromatographs on the (OH)Q[7]/PDMS stationary phase (Figure 4A) show slight peak tailing, suggesting alcohols may have stronger interactions with the stationary phase *via* H-bonding. However, sharp and symmetrical peaks for the same alcohols were obtained on the blank sol–gel PDMS column (Figure 4B), indicating a high inertness for the blank sol–gel PDMS column due to the inherent sol–gel column technology (Shende et al., 2003). Thus, slight peak tailing on the (OH)Q[7]/PDMS stationary phase may result from H-bonding interactions between the hydroxyl groups of alcohols and the carbonyl groups of (OH)Q[7], not from alcohols interacting with residual silanol on the stationary phase, which agrees well with its higher Y′ value in McReynolds' constants. Apart from H-bonding, a shape-fitting interaction between analytes and the cavity of (OH)Q[7] may also play a role in GC separation. To be more specific, the elution order for the pair of cyclohexanone and 2-heptanone was contrary to their boiling point order, that is, cyclohexanone (b.p. 156°C eluted before 2-heptanone (b.p. 152°C). The reason for this may be due to the molecular geometry of 2-heptanone probably fitting better with the cavity of (OH)Q[7] than that of cyclohexanone. These results verified that (OH)Q[7] on the stationary phase makes a great contribution to the separation performance of the (OH)Q[7]/PDMS column and makes the (OH)Q[7]/PDMS stationary phase exhibit good selectivity and excellent separation efficiency.

**Figure 5:**
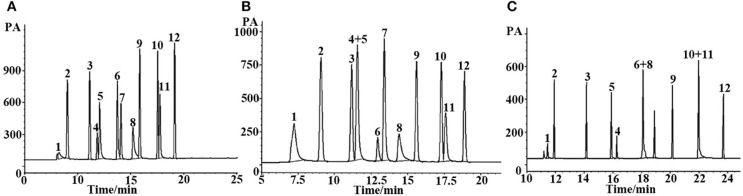
GC separation of the Grob mixture on **(A)** (OH)Q[7]/PDMS, **(B)** sol-gel blank PDMS and **(C)** commercial OV-1701 columns. Peaks: (1) 2,3-butanediol, (2) *n*- decane, (3) n- undecane, (4) nonanal, (5)1-octanol, (6) 2,6-dimethylphenol, (7) 2,6- dimethylaniline, (8) 2-ethylhexanoic acid, (9) methyl decanoate, (10) methyl undecanoate, (11) dicyclohexylamine, (12) methyl dodecanoate. Temperature program: 40°C (1 min) to 250°C at 5 °C/min.

Grob reagent is composed of 12 compounds with different polarities and structures, and is recognized as a test mixture for the comprehensive assessment of separation performance of a GC column as well as a chromatographic system. Resolution, elution order and peak shape of the analytes in the mixture reflect their retention behaviors on the given column as well as system conditions (Wang et al., 2014a). Figure 5 shows the chromatograms for separation of the Grob mixture on the (OH)Q[7]/PDMS, blank sol–gel PDMS and commercial columns. As shown in Figures 5A,B, the (OH)Q[7]/PDMS stationary phase generally achieved baseline resolution for almost all the compounds whereas the blank sol–gel PDMS column failed to separate nonanal from 1-octanol (Peaks 4, 5), which demonstrates that the addition of (OH)Q[7] to the stationary phase may increase the separation efficiency of the prepared column. In addition, two pairs of analytes (Peaks 6–8, Peaks 10, 11) were coeluted from the commercial OV-1701 column (Figure 5C). Compared with the reported neat Q[7], Q[8], or [OH]_12_Q[6] stationary phases, the pair of nonanal and 1-octanol or the pair of methyl undecanoate and dicyclohexylamine were coeluted from Q[7], Q[8], or [OH]_12_Q[6] columns (Sun et al., 2014, 2015; Zhang et al., 2014), demonstrating their lower separation performance than the (OH)Q[7]/PDMS column. Furthermore, compared with the reported ionic liquid sol–gel Q[6]-GBIL column or Q[7]-SG column, the (OH)Q[7]/PDMS column achieved much higher resolution for nonanal and 1-octanol (Peaks 4, 5) (*R* = 2.0) in the Grob mixture than the Q[6]-GBIL column (*R* = 1.0) (Wang et al., 2014a), and also obtained better separation efficiency between 2-ethylhexanoic acid, 2,6-dimethylphenol, methyl dodecanoate and 2,6-dimethylaniline than that of the reported Q[7]-SG column (Wang et al., 2014b). From an elution perspective, the (OH)Q[7]/PDMS stationary phase exhibited different retention behaviors from either the Q[6]-GBIL or the Q[7]-SG stationary phase due to their different sol–gel structures (Wang et al., 2014a,b). However, the elution order for all Grob mixtures on the (OH)Q[7]/PDMS stationary phase is the same as that of reported neat Q[7] column (Zhang et al., 2014), indicating the retention mechanism on the two stationary phases could be mainly based on molecular recognition of Q[7] toward analytes. This also verified that the preparation of the sol–gel (OH)Q[7]/PDMS stationary phase is successful, in which (OH)Q[7] is incorporated into the coating structure and gives the coating more integrity, and achieves better separation efficiency than that of the reported neat Q[7] column. In brief, the (OH)Q[7]/PDMS column showed advantages over the reported columns based on Q[n]s.

#### Separation of Mixtures With Similar Boiling Points

**Figure 6:**
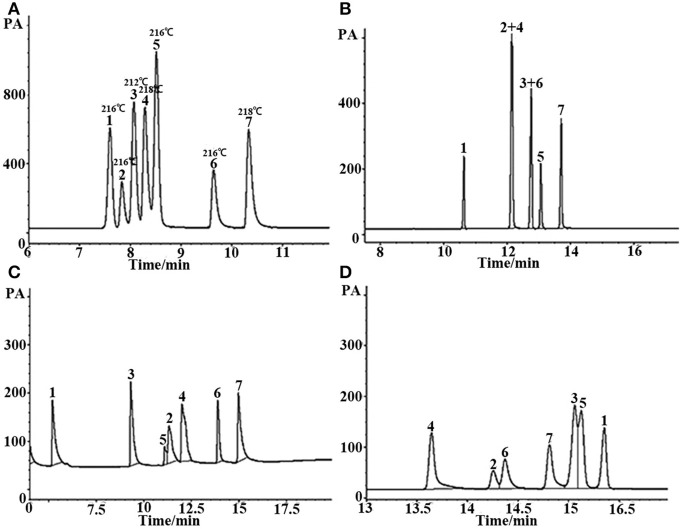
GC separation of the mixture with similar boiling point on **(A)** (OH)Q[7]/PDMS, **(B)** commercial OV-1701, **(C)** commercial FFAP, and **(D)** commercial SE-54 columns. Peaks: (1) *n*- dodecane (boiling point: 216°C), (2) *o*-nitrophenol (boiling point: 216°C), (3) ethyl benzoate (boiling point: 212°C), (4) phenethyl alcohol (boiling point: 218°C), (5) 2,6- dimethylaniline (boiling point: 216°C), (6) 2,6-dimethylphenol (boiling point: 216°C), (7) *p*- ethyl phenol (boiling point: 218°C). Temperature program: 80°C (1 min) to 250°C at 8°C/min.

In GC analysis, the elution order of analytes is commonly in agreement with their boiling point. The closer the boiling points of a pair of substances is, the more difficult they are to separate using GC. Figure 6 is the separation of seven kinds of analytes with very similar boiling points on the (OH)Q[7]/PDMS and commercial columns. The boiling points of these substances differ by no more than 6°C, and some of them even have the same boiling points. As can be seen from Figure 6, the seven analytes were basically baseline resolved on a 10-m-long sol–gel (OH)Q[7]/PDMS column with good peak shape, however, not only non-polar commercial 30-m-long SE-54 column, medium polar 30-m-long commercial OV-1701 column but also polar 30-m-long commercial FFAP column all failed to separate completely every analyte. This finding further suggests that the (OH)Q[7]/PDMS column has its distinctive separation characteristics. (OH)Q[7] on the stationary phase makes a great contribution to the separation of different analytes via H-bonding, fitness of cavity of (OH)Q[7], dipole–dipole interaction and even outer-surface interaction of (OH)Q[7] (Chen et al., 2014; Ni et al., 2014). The distinctive separation characteristics of (OH)Q[7]/PDMS also led to the elution order of seven analytes different from commercial columns, in which the elution order of analytes mainly depends on the polarity of the column and the polarity of the analyte itself.

#### Separation of Aromatic Geometric Isomers

**Figure 7:**
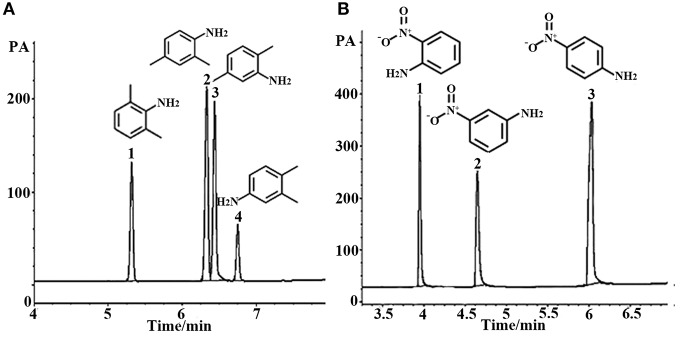
GC separation of dimethyl aniline on the (OH)Q[7]/PDMS column **(A)**. Peaks: (1) 2,6- dimethyl aniline, (2) 2,4- dimethyl aniline, (3) 2,5- dimethyl aniline, (4) 3,4- dimethyl aniline. Temperature program: 100°C (1 min) to 220°C at 8°C/min; GC separation of nitroaniline on the (OH)Q[7]/PDMS column at 100°C **(B)**. Peaks: (1) *o*- nitroaniline, (2) *m*- nitroaniline, (3) *p*- nitroaniline.

**Figure 8:**
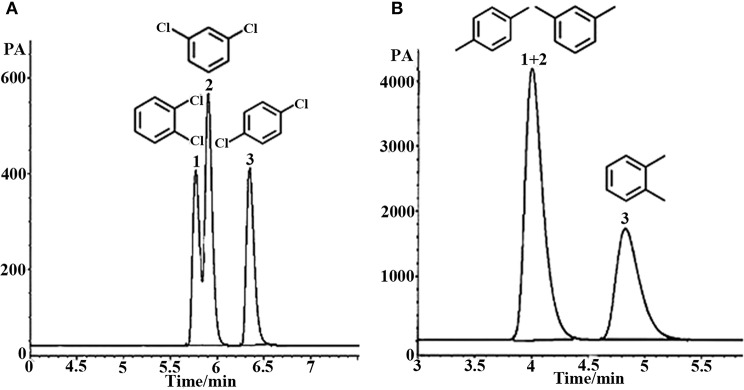
GC separation of dichlorobenzene on the (OH)Q[7]/PDMS column at 100°C **(A)**. Peaks: (1) *o*- dichlorobenzene, (2) *m*- dichlorobenzene, (3) *p*- dichlorobenzene; GC separation of xylene on the (OH)Q[7]/PDMS column at 70°C **(B)**. Peaks: (1) *p*-xylene, (2) *m*- xylene, (3) *o*-xylene.

Aromatic polar isomers and aromatic non-polar isomers were used to evaluate the selectivity and separation performance for the sol–gel-coated (OH)Q[7]/PDMS column. The results are shown in Figures 7, 8, Figures S1, S2, and Table 3. Figure 7, Figure S1, and Table 3 give the highly efficient separation of dimethylaniline, nitroaniline, benzenediol, and nitrophenol isomers (the resolution values *R* ranged from 2.01 to 8.92), demonstrating high selectivity of the newly developed sol–gel (OH)Q[7]/PDMS stationary phase toward these isomeric polar compounds. However, the (OH)Q[7]/PDMS stationary phase exhibited lower separation performance toward aromatic non-polar isomers, in which three kinds of non-polar isomeric compounds including dichlorobenzene isomers, the pair of phenanthrene and anthracene and xylene isomers were not separated completely from each other (Figure 8, Figure S2, and Table 3), even *p*-xylene and *m*-xylene were coeluted from the column. As mentioned above, the (OH)Q[7]/PDMS stationary phase could provide a greater interaction mode toward aromatic polar isomers, such as H-bonding, fitness of the cavity of (OH)Q[7], dipole–dipole interaction and dispersion forces, however, fitness of the cavity of (OH)Q[7] and dispersion force may be the main interactions between the stationary phase and aromatic non-polar isomers. Thus, the selectivity and separation performance of (OH)Q[7]/PDMS stationary phase for polar isomers may be better than that of non-polar isomers.

**Table 3:** Separation of geometric isomers of aromatic compounds on the sol-gel coated (OH)Q[7]/PDMS column.

**Solutes**	**Peak order**	**Resolution**			**Retention factor**				**Separation factor**		
		***R*_**1, 2**_**	***R*_**2, 3**_**	***R*_**3, 4**_**	***k*_**1**_**	***k*_**2**_**	***k*_**3**_**	***k*_**4**_**	**α_1, 2_**	**α_2, 3_**	**α_3, 4_**
Dimethyl aniline	2,6-, 2,4-, 2,5-, 3,4-	8.08	2.09	2.92	7.52	8.93	9.30	9.77	1.18	1.04	1.05
Nitroaniline	*o, m, p*	6.84	8.92		5.11	6.31	8.52		1.23	1.35	
Nitrophenol	*m, o, p*	2.97	2.01		3.62	6.44	6.85		1.78	1.06	
Benzenediol	*o, p, m*	6.83	2.66		8.25	10.77	11.82		1.30	1.09	
Dichlorobenzene	*m, p, o*	1.26	2.45		8.07	8.41	9.09		1.04	1.08	
Xylene	*p, m, o*	-	3.68		5.35	5.35	6.65		-	1.24	
phenanthrene and anthracene	Phenanthrene, anthracene	0.92			17.44	18.03			1.03		

### Thermodynamic Parameters for the (OH)Q[7]/PDMS Stationary Phase

The enthalpy change (Δ*H*) and entropy change (Δ*S*) were investigated to probe the energy effect on the retention behaviors of the (OH)Q[7]/PDMS stationary phase, which were calculated according to the van't Hoff equation:

$$\begin{array}{l} \text{ln }k=-\frac{\Delta H}{RT}+\frac{\Delta S}{R}+\text{ln }\beta \end{array}$$

where *k* is the temperature-dependent retention factor, *T* is the absolute temperature, *R* is the gas constant, and β is the phase ratio.

At each of the isothermal temperatures, the retention factor *k* of a given analyte can be determined. As such, the Δ*H* and Δ*S* for each analyte can be calculated on the basis of the linear relationship of ln *k* vs. 1/*T*. Two classes of analytes, namely polyaromatic hydrocarbons (PAHs) and alcohols, were used to perform isothermal separations at different temperatures over the range of 215–235 and 110–130°C in 5°C increments, respectively. The values of Δ*H* and Δ*S* for all the analytes are listed in Table 4, and corresponding van't Hoff plots are presented in Figure S3. As shown in Figure S1, all the coefficients of determination (*R*^2^) are larger than 0.99, demonstrating that there is a good linear relationship between ln *k* and 1/*T* for a given analyte. The ratio Δ*S/*Δ*H* is decreased for the separation of both alcohols and PAHs on the (OH)Q[7]/PDMS column, indicating that Δ*H* is the main driving force for the separation (Sun et al., 2013). Compared with a commercial OV-1701 column, the (OH)Q[7]/PDMS column showed lower Δ*S/*Δ*H* ratios for the same analytes, suggesting the higher selectivity of the latter. In general, more positive (or less negative) Δ*S* and more negative Δ*H* values are thermodynamically favorable for the transfer of the analyte from the mobile phase to the stationary phase, thus stronger retention for the analyte on the stationary phase (Ni et al., 2014).

**Table 4:** Values of Δ*H*, Δ*S*, Δ*S/*Δ*H* for separation of the indicated analytes on (OH)Q[7]/PDMS and commercial OV-1701 columns.

**Analyte**	**(OH)Q[7]/PDMS**				**OV-1701**			
	**-ΔH (KJ/mol)**	**-Δ*S* (J/mol K)**	**Δ*S*/Δ*H* (10^**−3**^/K)**	**R^**2**^**	**-Δ*H* (KJ/mol)**	**-Δ*S* (J/mol K)**	**Δ*S*/Δ*H* (10^**−3**^/K)**	**R^**2**^**
Naphthalene	41.4 ± 0.2	84.2 ± 0.5	2.0	0.995	44.1 ± 0.1	108.4 ± 0.2	2.5	0.998
Acenaphthene	47.2 ± 0.1	86.9 ± 0.2	1.9	0.998	53.3 ± 0.1	117.9 ± 0.3	2.2	0.996
Fluorene	48.0 ± 0.07	88.0 ± 0.2	1.8	0.999	57.6 ± 0.05	122.2 ± 0.1	2.1	0.999
Fluoranthene	53.9 ± 0.1	96.7 ± 0.2	1.7	0.999	66.0 ± 0.1	130.4 ± 0.2	1.9	0.999
Phenanthrene	61.6 ± 0.09	101.6 ± 0.2	1.6	0.999	70.7 ± 0.2	130.8 ± 0.5	1.8	0.996
1-Pentanol	31.5 ± 0.2	78.5 ± 0.5	2.5	0.997	39.5 ± 0.08	118.0 ± 0.2	3.0	0.999
1-Hexanol	39.9 ± 0.1	95.3 ± 0.3	2.4	0.999	38.6 ± 0.1	110.6 ± 0.2	2.9	0.999
1-Octanol	53.7 ± 0.1	120.5 ± 0.4	2.3	0.998	52.9 ± 0.2	138.8 ± 0.5	2.6	0.998
1-Nonanol	57.7 ± 0.1	126.0 ± 0.2	2.2	0.999	55.7 ± 0.3	140.9 ± 0.5	2.5	0.998
1-Decanol	64.3 ± 0.2	137.9 ± 0.4	2.1	0.998	61.6 ± 0.1	151.9 ± 0.2	2.4	0.999

## Conclusion

Q[7] was derivatized to (OH)Q[7] and coupled with KH-560 to be immobilized onto the inner wall of a capillary column by sol–gel technology. The presented method successfully addresses and eliminates some of the drawbacks associated with reported stationary phases based on Q[n]s. Such shortcomings include low column efficiency and low selectivity resulting from Q[n] not dissolving in organic solvents or Q[n] only encapsulated in the stationary phase. The proposed (OH)Q[7]/PDMS stationary phase gives full play to the unique nature of (OH)Q[7] and demonstrates high selectivity and good resolution toward a wide range of analytes, which shows great potential for application in the science of separation. However, some critical pairs in GC such as xylene isomers still do not provide a good separation performance on the proposed (OH)Q[7]/PDMS stationary phase, which will prompt us to do further research work.

## Data Availability Statement

All datasets generated for this study are included in the article/Supplementary Material.

## Author Contributions

JH, JR, JY, LZ, SW, and YW contributed conception and design of the study. JH and JR organized the database. LZ and SW performed the statistical analysis. ND wrote the first draft of the manuscript. JH and YW wrote sections of the manuscript. All authors contributed to manuscript revision, read, and approved the submitted version.

### Conflict of Interest

The authors declare that the research was conducted in the absence of any commercial or financial relationships that could be construed as a potential conflict of interest.
