# Supplementary material for: Separation Performance of Capillary Gas Chromatography Based on Monohydroxycucurbit[7]Uril Incorporated Into Sol–Gels as the Stationary Phase
Source: Front Chem. 2020 Feb 5;8:31. doi: 10.3389/fchem.2020.00031 (PMC7014919; doi:10.3389/fchem.2020.00031)
Supplement: Supplementary file 1 [file Table_1.doc]

**Electronic Supplementary Material for**

**Separation performance of capillary gas chromatography based on monohydroxycucurbit[7]uril incorporated into sol–gels as the stationary phase**

**Jing He^a^, Jingfeng Ran^a^, Jianmei Yao^a^, Lingxue Zhang^a^, Shasha Wang^a^, Yuan Wang^c^, Nan Dong^a,b,*^**

**^a^School of Chemistry and Chemical Engineering, Guizhou University, Guiyang, 550025, China**

**^b^Key Laboratory of Macrocyclic and Supramolecular Chemistry of Guizhou Province, Guiyang, 550025, China**

**^c^Resource and Environmental Engineering College, Guizhou University, Guiyang, 550025, China**

1. **Chemical ingredients of the coating**

The chemical ingredients used to create the sol–gel Q7OH/PDMS coating are presented in Table S1.

**Table S1** Names, functions and chemical structures of sol-gel Q7OH/PDMS coating ingredients

| The name of analytes | Function | The structure of analytes |
| --- | --- | --- |
| OH-PDMS | Coating stationary phase |  |
| Q7OH | Coating stationary phase | 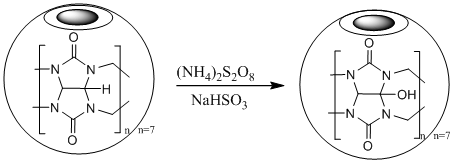 |
| KH-560 | Sol-gel precursor |  |
| MTMOS | Sol-gel precursor |  |
| PMHS | Deactivation reagent |  |
| TFA | Acid catalyst | CF_3_COOH |

**2. The possible structure of coating**

A simplified scheme of the sol–gel Q7OH/PDMS coating on the fused-silica capillary inner wall is shown in Scheme S1.


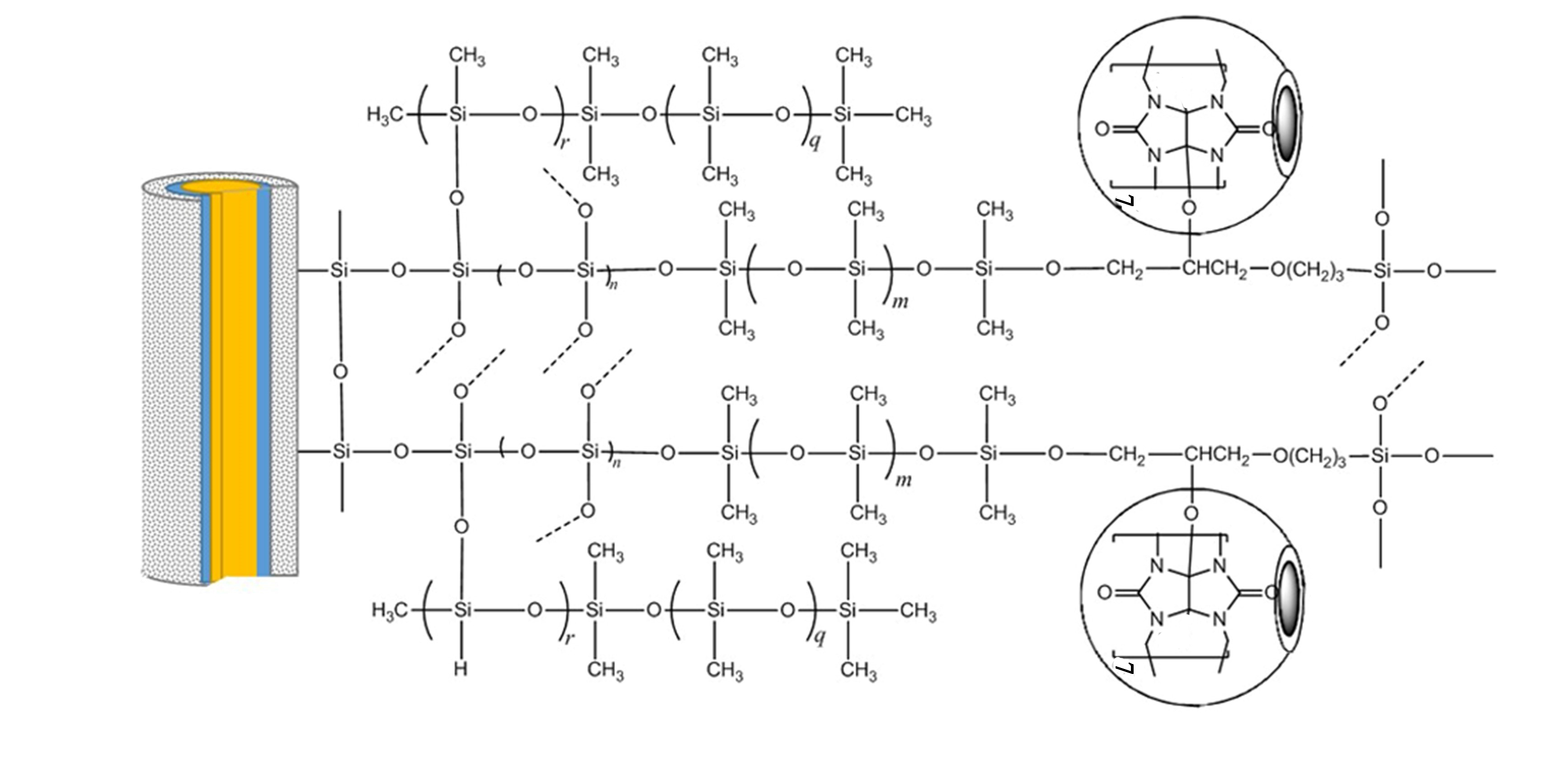


**Scheme S1** The possible structure of the coating

**3. McReynolds constants of the Q7OH/PDMS column**

Five probe compounds, namely benzene, *n*-butanol, 2-pentanone, nitropropane and pyridine, are used for the determination of McReynolds’ constants of the prepared column. The results are shown in Table S2. The Q7OH/PDMS column had an average polarity of 143 and can be classified as weakly-to-moderately polar nature in GC separation.

**Table S2** McReynolds constants for the sol-gel Q7OH/PDMS and commercial capillary columns

| Column | X′ | Y′ | Z′ | U′ | S′ | Sum | Average |
| --- | --- | --- | --- | --- | --- | --- | --- |
| *Ι* for squalene | 653 | 590 | 627 | 652 | 699 |  |  |
| *I* for Q7OH/PDMS | 711 | 851 | 729 | 803 | 839 |  |  |
| *I* for SE-54 | 684 | 783 | 690 | 721 | 857 |  |  |
| *I* for OV-1701 | 728 | 1178 | 779 | 887 | 842 |  |  |
| △*I* for Q7OH/PDMS | 58 | 261 | 102 | 151 | 140 | 715 | 143 |
| △*I* for SE-54 | 31 | 193 | 63 | 69 | 94 | 450 | 90 |
| △*I* for OV-1701 | 75 | 588 | 152 | 235 | 143 | 1193 | 238 |

X′: benzene, Y′ : 1-butanol, Z′ : 2- pentanone, U′ : 1- nitropropane, S′: pyridine. Temperature: 120℃

**4. Solvent stability of the sol-gel Q7OH/PDMS column**

The solvent stability was tested by comparing the solute retention times in five replicates before and after rinsing the column with methylene chloride. The results are presented in Table S3. As shown in Table S3, The data presented in Table S3 suggest that the solute retention time does not change obviously, which showed that this column has excellent solvent stability.

**Table S3**

Retention time repeatability for the prepared test mixture before and after rinsing column with methylene chloride (n=5) ^a^

| Solute | Before rinsing (average) | | After rinsing (average) | |
| --- | --- | --- | --- | --- |
|  | Retention time (min) | RSD (%) | Retention time (min) | RSD (%) |
| 2- Pentanone | 2.72 | 0.42 | 2.88 | 0.37 |
| Pentanol | 5.98 | 0.52 | 6.31 | 0.54 |
| *n*- Decane | 9.52 | 0.21 | 9.87 | 0.08 |
| *n*- Undecane | 11.92 | 0.37 | 12.28 | 0.20 |
| *n*- Octanol | 13.68 | 0.32 | 14.08 | 0.52 |
| 2,6- Dimethylphenol | 15.30 | 0.43 | 15.67 | 0.19 |
| Methyl decanoate | 17.44 | 0.09 | 17.74 | 0.81 |
| Methyl undecanoate | 19.33 | 0.18 | 19.96 | 0.17 |
| Methyl dodecanoate | 21.06 | 0.09 | 21.43 | 0.15 |
| *p*- Nitrophenol | 28.30 | 0.02 | 28.54 | 0.12 |

^a^ Conditions: column, 10 m × 0.25 mm i.d. fused- silica capillary column; stationary phase, sol-gel Q7OH/PDMS; split (30:1, 250℃); detector, 300℃; column temperature programmed from 70 ℃ at 5 ℃ min^-1^

**5. Separation of aromatic geometric isomers**

Aromatic isomers benzenediol , nitrophenol , phenanthrene and anthracene were separated on the (OH)Q[7]/PDMS column and their chromatography is presented in Figure S2 and Figure S3. As shown in Figure S2, polar isomers benzenediol and nitrophenol can be achieved baseline resolution; however, nonpolar isomers phenanthrene and anthracene are not separated completely from each other (Figure S3).


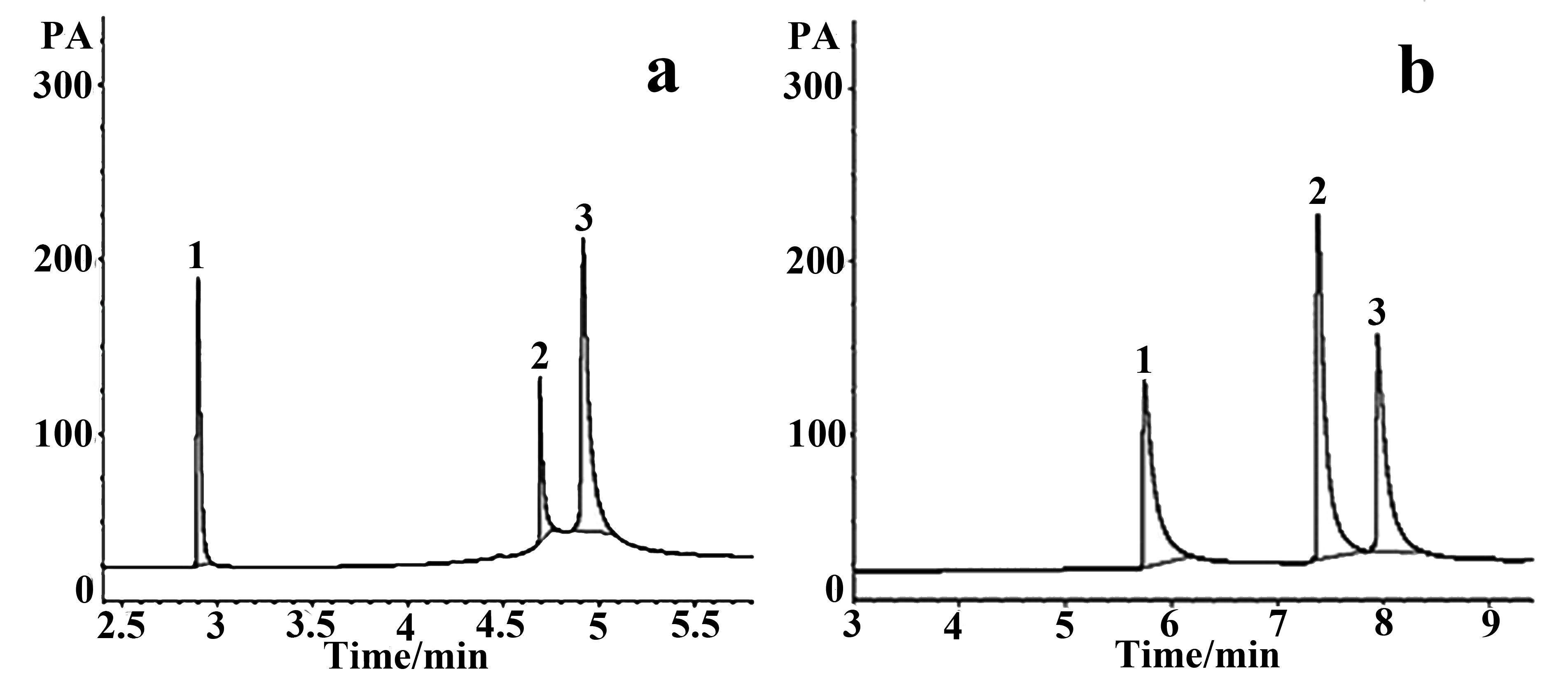


**Figure S1** GC separation of nitrophenol on the Q7OH/PDMS column at 120 ℃ (a). Peaks: (1) *m*- nitrophenol, (2) *o*- nitrophenol, (3) *p*- nitrophenol ; GC separation of benzenediol on the Q7OH/PDMS column at 120 ℃ (b). Peaks: (1) *o*- benzenediol, (2)*p*- benzenediol, (3) *m*- benzenediol


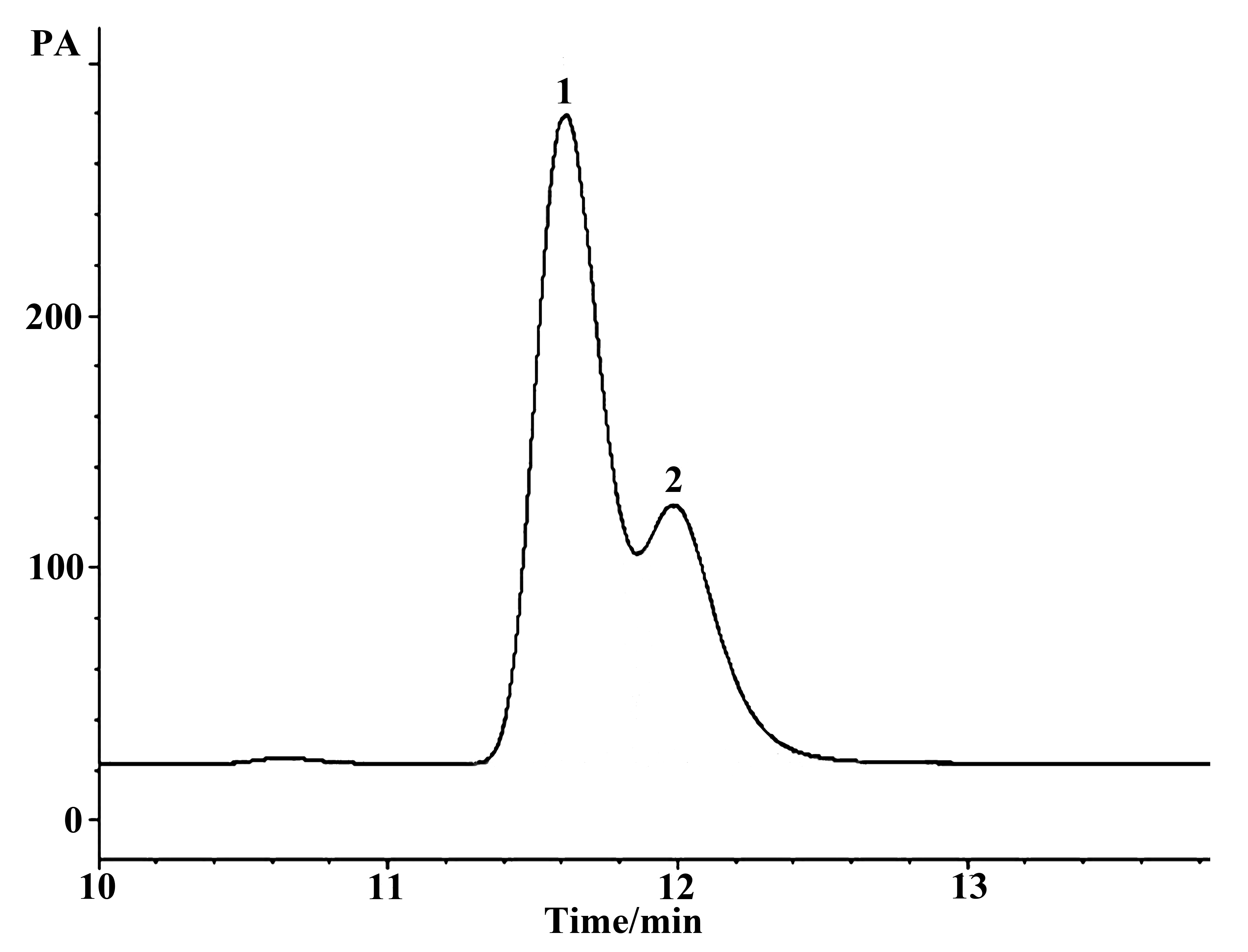


Figure S2 GC separation of phenanthrene and anthracene on the Q7OH/PDMS column at 180 ℃. Peaks: (1) phenanthrene, (2) anthracene

**6. Van’t Hoff curves on the Q7OH/PDMS capillary column**

The temperature dependence of the retention factor *k* can be described by the van’t Hoff equation. The related plot between ln *k* and 1/*T* is shown in Figure S1. As shown in Figure S1, the coefficient of determination (*R*^2^) is larger than 0.99 for all the analytes, demonstrating the good linearity of ln *k* and 1/*T* for a given analyte.


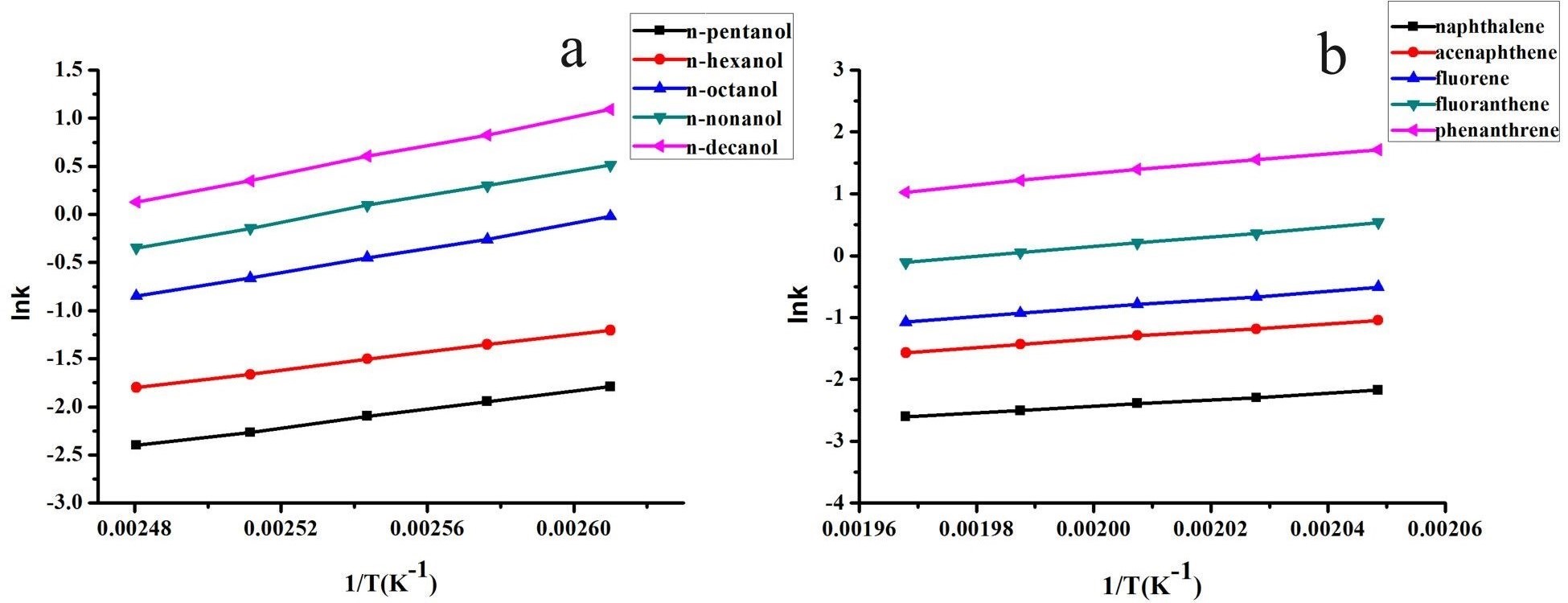


**Figure S3** Van’t Hoff curves for (a) PAHs and (b) alcohols on the Q7OH/PDMS capillary column
